# Additions and corrections to the check list of the Noctuoidea (Insecta, Lepidoptera) of North America north of Mexico IV

**DOI:** 10.3897/zookeys.252.28500

**Published:** 2018-10-08

**Authors:** B. Christian Schmidt, J. Donald Lafontaine, James T. Troubridge

**Affiliations:** 1 Canadian National Collection of Insects, Arachnids, and Nematodes, Biodiversity Program, Agriculture and Agri-Food Canada, K.W. Neatby Bldg., 960 Carling Ave., Ottawa, Ontario, Canada K1A 0C6 Canadian National Collection of Insects, Arachnids, and Nematodes Ottawa Canada; 2 Hagersville, Ontario, Canada Unaffiliated Hagersville Canada

**Keywords:** Canada, United States, Erebidae, Noctuidae, Nolidae

## Abstract

A summary of all taxonomic and nomenclatural changes to the check list of the Noctuoidea of North America north of Mexico since the last update published in 2015 is provided. A total of 64 changes are listed and discussed, consisting of 26 recently published changes and additions, and an additional 38 presented herein. One stat. n., one stat. rev., six syn. n., and two comb. n. are proposed for the first time. *Orthimella* Schmidt & Lafontaine nom. n. is proposed here as an objective replacement name for *Himella* Grote, 1874 [Noctuinae: Orthosiini], a junior homonym of *Himella* Dallas, 1852 [Hemiptera: Coreidae].

## Introduction

Continuing work on the taxonomy and systematics of New World Noctuoidea has resulted in 64 additional changes to the check list of North American Noctuoidea (Lafontaine and Schmidt 2010). In terms of the North American fauna diversity, the current work summarizes changes with 43 species-level taxa added and nine deleted for a net gain of 34. These are in addition to the 115 changes made in 2011 (Lafontaine and Schmidt 2011), and 64 made in 2013 (Lafontaine and Schmidt 2013), and the 124 made in 2015 (Lafontaine and Schmidt 2015). The new total for Noctuoidea in North America north of Mexico is 3706 species.

## Repository abbreviations

Taxonomic changes are based on examination of material, especially type specimens, in the following collections:

**AMNH** The American Museum of Natural History, New York, NY, USA

**ANSP** The Academy of Natural Sciences, Philadelphia, Pennsylvania, USA

**NHMUK** The Natural History Museum, London, UK

**CNC** Canadian National Collection of Insects, Arachnids, and Nematodes, Ottawa, Ontario, Canada

**CU** Cornell University Insect Collection, Ithaca, New York, USA

**KBC** Knudson/Bordelon Collection at MCLB – McGuire Center for Lepidoptera and Biodiversity, University of Florida, Gainesville, FL, USA

**MNHN** Muséum National d’ Histoire Naturelle, Paris, France

**MSU** Michigan State University, East Lansing, Michigan, USA

**TLSRC** Texas Lepidoptera Survey Research Collection, Houston, Texas, USA

**JTTC** James T. Troubridge Collection, Hagersville, Ontario, Canada

**USNM** National Museum of Natural History [formerly, United States National Museum], Washington, District of Columbia, USA

## Results

### Corrections, additions, and changes (highlighted in bold)

**930207.1 *Hypoprepialampyroides* Palting & Ferguson, 2018**

**930215.1 *Clemensiaumbrata* Packard, 1872**

**930216 *Clemensiaochracea* Schmidt & Sullivan, 2018**

(= *Clemensiapatella* of authors)

**930233 – 930238 *Chelis* Rambur, 1866**

**(= *Holoarctia, Neoarctia*, *Hyperborea*)**

**930239 – 930277** *Apantesis*

**(= *Holarctia, Grammia, Notarctia*)**

**930283 – 930289** *Arctia*

**(= *Parasemia, Acerbia, Pararctia, Platarctia, Platyprepia*)**

**930299.1 *Virbiamarginata* (Druce, 1885)**

**930409.1 *Euchaetesnancyae* Nagle & Schmidt, 2018**

**930468.1 *Nyridelaxanthocera* (Walker, 1856)**

**930541.1 *Thursanialycimnia* (Druce, 1891)**

**930589.1 *Hypenaopulenta* (Christoph, 1877)**

**930714.1 *Glympisholothermes* Hampson, 1926**

**930723.1 *Nychiopterabasipallida* Barnes & McDunnough, 1916**

930730 ***Hyperstrotiavillificans* (Barnes & McDunnough, 1918)**

**930822.1 *Catocalaventura* Borth & Kons, 2016**

**930829.1 *Catocalaslotteni* Kons & Borth, 2016**

**930834.1 *Catocalabastropi* Kons & Borth, 2017**

**930887.1 *Buliamexicana* (Behr, 1870)**

930913 *Drasteriahowlandii* (Grote, 1865)

**syn. *D.tejonica* (Behr, 1870)**

**930968.11 *Mimophismaforbesi* Schaus, 1940**

**930969.1 *Tyrissarecurva* Walker, 1866**

**931000.1 *Toxonpruchascitior* (Walker, 1865)**

931019 ***Zalestrigimacula* (Guenée, 1852)**

931020 **Delete***Zaleobsita* (Guenée, 1852)

**931071.1 *Eulepidotismerricki* (Holland, 1902)**

931143 *Nycteolacolumbiana* (H. Edwards, [**1874**])

931273 **Delete***Cobubathaochrocraspis* Hampson, 1910

931278 **Delete***Cobubathahippotes* (Druce, 1889)

**931290 *Protodeltote* Ueda, 1984**

**931540.1 *Dolocuculliapoolei* Crabo & Hammond, 2018**

**931659.1 *Plagiomimicusyakama* Crabo & Wikle, 2018**

**ssp. *P.y.yakama* Crabo & Wikle, 2018**

**ssp. *P.y.mojave* Wikle & Crabo, 2018**

931719 *Neogrotella****mcdunnoughi*** Barnes & Benjamin, 1922

**syn. *N.macdunnoughi*, misspelling**

**931815.1 *Sympistiseleaner* Adams, 2018**

**931821.1 *Sympististenuistriga* (McDunnough, 1940)**

**931927.1 *Sympistisferrirena* Crabo, 2018**

**931970.1 *Neotuertacollectiora* Todd, 1966**

**932029.1 *Diastemachuza* (Druce, 1898)**

**932045.1 *Helicoverpaarmigera* (Hübner, [1808])**

**932130.1 *Schiniaamblys* (Dyar, 1913)**

932139 *Schiniavolupia* (Fitch, 1868)

**syn. *S.masoni* (Smith, 1896)**

**932225.1 *Elaphriahypophaea* (Hampson, 1920)**

932587 *Eupsiliavinulenta* (Grote, 1864)

**syn. *E.walkeri* (Grote, 1864)**

**932588.1 *Eupsiliacolorado* (Smith, 1903)**

**932588.2 *Eupsiliaschweitzeri* Lavitt & Wagner, 2016**

**932606.1 *Chaetaglaearhonda* Stead & Troubridge, 2016**

**932615.1 *Omphaloscelislunosa* (Haworth, [1809])**

932643 *Aseptissusquesa* (Smith, 1908)

**syn. *A.monica* (Barnes & McDunnough 1918)**

**932645.1 *Aseptisharpi* Crabo & Mustelin, 2018**

932656 *Stretchia****plusiaeformis*** H. Edwards, 1874

**syn. *S.plusiiformis*, misspelling**

932781 *Orthosiatenuimacula* (Barnes & McDunnough, 1913)

**syn. *O.mediomacula*** Barnes & McDunnough, 1924

**syn. *O.nongenerica*** Barnes & McDunnough, 1924

932806 **move to 2924.50**

932807 **move to 2924.52**

**932785 *Orthimella****fidelis* (Grote, 1874) **comb. n.**

**932924.50***Admetovisoxymorus* Grote, 1873 **moved from 932806**

**932924.51 *Admetovisicarus* Crabo & Schmidt, 2018**

**932924.52***Admetovissimilaris* Barnes, 1904 **moved from 932807**

**932937.1 *Leucaniaclarescens* Möschler, 1890**

**932953.1***Leucaniaoregona* Smith, 1902

**932953.2 *Leucaniachejela* (Schaus, 1921)**

**932955.1 *Leucaniarawlinsi* Adams, 2001**

**932961.1 *Leucanialatiuscula* Herrich-Schäffer, 1868**

**933115.1 *Rhabdorthodespattersoni* Crabo, 2018**

**933181.1 *Hypotrixlactomellis* Wikle & Crabo, 2018**

933207 *Hydroeciodesserrata* (Grote, 1880)

**syn. *H.ochrimacula* (Barnes & McDunnough, 1913)**

**933663.1 *Abagrotisbenjamini* Franclemont, 1955**

### Notes

**93027.1 *Hypoprepialampyroides*** – This species is described in the current volume by Palting et al. (2018).

**930215.1 *Clemensiaumbrata*** – This species is raised from synonymy by Schmidt and Sullivan (2018).

**930216 *Clemensiaochracea*** – This species is described in the current volume by Schmidt and Sullivan (2018).

**930233 – 930238 *Chelis*** – The genera *Holoarctia*, *Neoarctia*, and *Hyperborea* were subsumed under *Chelis* by Rönkä et al. (2016).

**930239 – 930277 *Apantesis*** – The genera *Holarctia*, *Grammia*, and *Notarctia* were subsumed under *Apantesis* by Rönkä et al. (2016).

**930283 – 930289 *Arctia*** – The genera *Parasemia*, *Acerbia*, *Pararctia*, *Platarctia*, and *Platyprepia* were subsumed under *Arctia* by Rönkä et al. (2016).

**930299.1 *Virbiamarginata*** – the *Virbia* taxon from southernmost Texas previously thought to be *V.aurantiaca* is in fact more closely related to *V.marginata* (described from Guatemala) based on phenotype and DNA barcode, and this species is accordingly added to the North American fauna. The western species treated as *V.marginata* by Zaspel et al. (2008) is a separate, undescribed species maintained as Virbianearmarginata, as per Lafontaine and Schmidt (2010).

**930409.1 *Euchaetesnancyae*** – This species is described in the current volume by Nagle and Schmidt (2018).

**930468.1 *Nyridelaxanthocera*** – reported and photographed in southern Texas in 2017 (Krancevic 2018).

**930541.1 *Thursanialycimnia*** – Several specimens of this Mexican species were collected in southern Texas in 2015 by Ed Knudson. Vouchers are in KBC.

**930589.1 *Hypenaopulenta*** – this species was approved for release in eastern Canada and is pending approval in the United States of America as a biological control agent of the invasive European swallow-worts (*Vincetoxicum* spp.). It has become established at several locations in eastern Ontario since 2016 (R. Bourchier, pers. comm.).

**930714.1 *Glympisholothermes*** – This species was found at Crocodile Lake National Wildlife Refuge in the Florida Keys by David Fine in 2009.

**930723.1 *Nychiopterabasipallida*** – This species was described in the genus *Oxycilla* Grote by Barnes and McDunnough (1916) and has remained there until now. The barcode results suggested the species belonged in the genus *Nychioptera* Franclemont in the Boletobiinae and not in *Oxycilla* in the Rivulinae and examination of the male genital characters confirmed the new placement as ***Nychiopterabasipallida*** (Barnes & McDunnough, 1916), **comb. n.**

**930730 *Hyperstrotiavillificans*** – This species was synonymized with *H.nana* (Hübner, 1818) by Lafontaine and Schmidt (2015). New barcode data has shown there are two similar species going under the name *H.nana*. Specimens of the more southern of the two species, with a range extending from Pennsylvania and Illinois southward to Florida, closely match the holotype of *H.villificans*. We therefore re-instate the name *Hyperstrotiavillificans*, **stat. rev.** for this taxon. The type specimen for *H.nana*, like those of most names published by Hübner, is lost or destroyed, but the rather schematic painting in Hübner (1818: 14, figs 53, 54) more closely resembles the more widespread species that occurs from southern Canada to Florida, so we apply the name *H.nana* to this species. A neotype should be selected for *H.nana* when the genus *Hyperstrotia* Hampson is revised.

**930822.1 *Catocalaventura*** – Described as being a new species similar to but distinct in appearance and barcodes from *Catocalacaliforniensis* Brower and *C.johnsoniana* Brower (Borth and Kons 2016).

**930829.1 *Catocalaslotteni*** – Described from the Florida Panhandle as a southern relict species related to *Catocalawhitneyi* Dodge from the northern prairies. Both species are associated with leadplant (*Amorpha* L., Fabaceae) (Kons and Borth 2016).

**930834.1 *Catocalabastropi*** – Described from western Louisiana and eastern Texas, this new species occurs west of the known range of *Catocalalouiseae* Bauer, which occurs as far west as Alabama (Kons and Borth 2017).

**930887.1 *Buliamexicana*** – Recently discovered to occur in southern Texas. One of the two vouchers in KBC has been barcoded.

**930913 *Drasteriahowlandii*** – Typical *Drasteriahowlandii* is a species of the Great Plains and western mountainous areas and is replaced farther south by a paler desert form in which females look like typical *D.howlandii* but males have more white in the hind wing. Richards (1939) treated them as separate species because their range only overlapped slightly, but he also pointed out that there were many exceptions to the “species” characters with wrong “forms” showing up in each other’s territory and suggested the two “species” hybridize where their ranges meet. The barcodes also do not match either distribution or color forms, and unlike other closely-related *Drasteria*, barcode variation is also very low; we therefore treat *D.tejonica* (Behr, 1870), **syn. n.** as a geographic form of *D.howlandii*.

**930968.11 *Mimophismaforbesi*** – This species was previously known only from Puerto Rico, but found in the Florida Keys by Jim Troubridge in 2013. The specimen has been barcoded.

**930969.1 *Tyrissarecurva*** – Specimens were collected at the National Key Deer Refuge in the Florida Keys by David Fine and Jim Troubridge.

**931000.1 *Toxonpruchascitior*** – This species was described from northwestern Guatemala but is now known to occur through Mexico and into the Hill Country of Texas. Vouchers are in the collection of Hugo Kons Jr., Florida, and the Biodiversity Institute of Ontario, University of Guelph, Ontario.

**931019 *Zalestrigimacula*** – The identity of this species has been a mystery for many years. It had been reported from Florida, but specimens from Florida identified as *Z.strigimacula* and those identified as *Z.viridans* (Guenée, 1852) were found to represent a single undescribed species unknown from the Neotropics or elsewhere in the Caribbean, so both species were removed from the check list of Canada and United States (Lafontaine and Schmidt 2010) in a recent list update (Lafontaine and Schmidt (2015). Unfortunately, the abdomen of the male lectotype borrowed from MNHP by J. G. Franclemont for dissection has been lost. However, Neotropical specimens in USNM dissected and identified as *Zalestrigimacula*, and presumably compared to the dissection of the lectotype, give a clue to the identity of this species. The species occurs from Brazil northward into southern Texas and is therefore placed back on the North American checklist; this species is, however, not known to occur in Florida. At least one additional species belonging to the *Z.strigimacula* complex is known from Texas.

**931020 *Zaleobsita*** – As with *Zalestrigimacula* above, there has been much confusion as to the correct identity of this species. Specimens identified as *Zaleobsita* from Florida are now reidentified as the same undescribed species discussed under *Z.strigimacula*, so this particular species has been the basis for the incorrect reports of *Zalestrigimacula*, *Z.obsita*, and *Z.viridans* from Florida. The female genitalia of the *obsita* type in the NHMUK is unique in having a single elongated lobe to the corpus bursae, unlike the figure 8-shaped bursa of species in the *Z.strigimacula* complex. Alberto Zilli pointed out that the genitalia of specimens from the Galapagos Islands and treated as *Z.obsita* by Hayes (1973) were good matches for the type specimen from Brazil. Dissection and barcoding of specimens from the Galapagos in the CNC confirms this identification and show that *Z.obsita* is known from Brazil, Ecuador, Venezuela, Costa Rica, and Guatemala, but not from farther north.

**931071.1 *Eulepidotismerricki*** – Specimens were collected at the National Key Deer Refuge in the Florida Keys by Jim Troubridge and David Fine. A specimen has been barcoded.

**931143 *Nycteolacolumbiana*** – This species was described in the Proceedings of the California Academy of Sciences, volume 5, 1873, with internal dates through the volume indicating the various fascicles within it were printed in various months of 1873. However, Poole (1989: 1039) and Nye (1975: 410 [under *Pseudalypia*]) give 1874 as the year of publication for this volume. The year used by Nye is based on the postal cancellation date in the library of NHMUK – the library being one of the few that save the postal wrappers. So the date is corrected here to 1874. There being no internal evidence of the Edwards paper being published in 1874, the corrected year is in brackets. Contributed by Lars Crabo.

**931273 *Cobubathaochrocraspis*** – This species was added to the North American list (Lafontaine and Schmidt 2010) on the basis of specimens identified as *Cobubathaochrocraspis* in USNM that closely resemble *Cobubathametaspilaris* Walker, 1863 from Florida and the Caribbean. Examination of the holotype in the NHMUK shows that the specimens in USNM were incorrectly associated with this name and are *Cobubathametaspilaris*, which is now known to occur in the United States in Florida, Texas, and Arizona. The holotype of *Cobubathaochrocraspis* belongs in the genus *Tripudia* Grote, as Poole (1989) correctly determined. *Tripudiaochrocraspis***comb. rev.** occurs from Jalapa in southern Mexico to Costa Rica.

**931278 *Cobubathahippotes*** – This species was described from Guatemala in 1889. It was reported as *C.hippotes* in the Noctuidae MONA check list (Franclemont and Todd 1983), but the species recorded in Texas is now known to be an undescribed species related to *C.hippotes*.

**931290 *Protodeltote*** – Both *Protodeltote* and *Deltote* were recognized as valid genera in Lafontaine and Schmidt (2010) following the revision by Ueda (1984), but at the time we were unaware that *Protodeltote* had recently been subsumed within *Deltote* as a subgenus by Fibiger et al. (2009). Despite the apparent similarity between the two genera, phylogenetic analysis shows that the two are in fact not closely related (BCS, unpubl. data), and we therefore re-instate *Protodeltote***stat. rev.** as a valid genus as proposed by Ueda (1984).

**931540.1 *Dolocuculliapoolei*** – This species is described in the current volume by Crabo et al. (2018).

**931659.1 *Plagiomimicusyakama*** – This species, with two constituent subspecies, is described in the current volume by Crabo et al. (2018).

**931719 *Neogrotellamcdunnoughi*** – The species name was misspelled as *macdunnoughi* following the spelling in Franclemont and Todd (1983). Contributed by Greg Pohl & Steve Nanz.

**931815.1 *Sympistiseleaner*** – This taxon is described in Adams and Schmidt (2018) in the current volume.

**931821.1 *Sympististenuistriga*** – Sympistisbadistrigavar.tenuistriga (McDunnough, 1940) was first treated as a valid species in Pohl et al. (2018) based on genital and barcode differences.

**931927.1 *Sympistisferrirena*** – This species is described in the current volume by Crabo et al. (2018).

**931970.1 *Neotuertacollectiora*** – This taxon was described as a Cuban subspecies of *Neotuertasabulosa* (Felder, 1874), a species mainly occurring in Central and South America and the Caribbean as far north as Puerto Rico. Research by Jim Troubridge indicates the Cuban taxon should be raised to species status as *Neotuertacollectiora* Todd, 1966, **stat. n.** It was collected at Crocodile Lake National Wildlife Refuge in the Florida Keys in 2016 by David Fine.

**932029.1 *Diastemachuza*** – *Diastemachuza* (Druce, 1898), **comb. n.** was included in the genus *Eustrotia* Hübner by Hampson (1910) and Poole (1989), but the barcodes and genitalia associate it with the genus *Diastema* Guenée. It has been found in Texas in Starr County.

**932045.1 *Helicoverpaarmigera*** – The Old World Bollworm, a significant pest species native to the eastern hemisphere, is now also established in South America. This species was detected in Florida (Manatee County: Bradenton) in 2015, but appears not to have become established (USDA 2017). This species has the potential to become an agricultural pest in North America (USDA 2017).

**932130.1 *Schiniaamblys*** – This mainly Mexican species has recently been found in southeastern Arizona (D. Wikle pers. comm.).

**932139 *Schiniavolupia*** – Synonymy with *Schiniamasoni* from Pogue et al. 2013.

**932225.1 *Elaphriahypophaea*** – Specimens of *Elaphriafuscimacula* (Grote, 1881) from southern Texas southward have been re-identified by JDL as the central and northern South American species *Elaphriahypophaea* on the basis of barcodes and differences in the male genitalia. *Elaphriafuscimacula* is a complex of three species that occur from Florida and North Carolina to central Texas. The type locality of *Monodesfuscimacula* Grote is Tallahassee, Florida.

**932587 *Eupsiliavinulenta*** – The name *Eupsiliawalkeri* (Grote, 1864) was transferred from the synonymy of *Eupsiliasidus* (Guenée, 1852) to the synonymy of *Eupsiliavinulenta* (Grote, 1864) by Lavitt and Wagner (2016).

**932588.1 *Eupsiliacolorado*** – This name was previously treated as a synonym of *Eupsiliasidus* (Guenée, 1852), but was raised to the status of a valid species by Lavitt and Wagner (2016). It occurs in southwestern Colorado, highly isolated from populations of *Eupsiliasidus* in eastern United States.

**932588.2 *Eupsiliaschweitzeri*** – This new species was initially distinguished from *E.sidus* by barcode and larval differences, but also differs in details of the male genitalia (Lavitt and Wagner 2016).

**932606.1 *Chaetaglaearhonda*** – This recently described species (Stead and Troubridge 2016) refers to populations from the Carolinas northward to southern Ontario previously identified as *Chaetaglaeatremula*.

**932615.1 *Omphaloscelislunosa*** – A European introduction first reported from North America at Potomac, Maryland, 7 October 2015, by Tomas Mustelin.

**932643 *Aseptissusquesa*** – The synonymy of *Aseptismonica* and *A.susquesa* by Mustelin and Crabo 2015 was in inadvertently missed in Lafontaine and Schmidt 2015.

**932645.1 *Aseptisharpi*** – This species is described in the current volume by Crabo et al. (2018).

**932656 *Stretchiaplusiaeformis*** – The species name *plusiaeformis* was incorrectly updated to *plusiiformis* by Lafontaine and Schmidt (2015) following Poole (1989). The correct original spelling is *plusiaeformis*. Contributed by Greg Pohl & Steve Nanz.

**932781 *Orthosiatenuimacula*** – Barcodes and dissections confirm that *O.mediomacula* Barnes & McDunnough, 1924, **syn. n.** and *O.nongenerica* Barnes & McDunnough, 1924, **syn. n.** are color forms of *Orthosiatenuimacula*.

**932785 *Orthimella* Schmidt & Lafontaine, nom. n.** is proposed here as an objective replacement name for *Himella* Grote, 1874 [Noctuinae: Orthosiini, type species *Himellafidelis* Grote, 1874], a junior homonym of *Himella* Dallas 1852 [Hemiptera: Coreidae], a valid genus of neotropical coreids. This action results in the following new combination: *Orthimellafidelis* (Grote, 1874) **comb. n.**

932806 – see entry for 2924.51

932807 – see entry for 2924.51

**932924.51 *Admetovisicarus*** – This species is described in the current volume by Crabo and Schmidt (2018). The genus Admetovis is re-classified as a member of the tribe Hadenini from its previous placement in the Orthosiini, resulting in the re-assignment of checklist numbers from 932806 – 2807.

**932937.1 *Leucaniaclarescens*** – This species was described from Puerto Rico. Jim Troubridge collected specimens at Bahia Honda State Park and Crocodile Lake National Wildlife Refuge in the Florida Keys and identification was made by Cliff Ferris from the male genitalia of one these specimens. The other specimen has been barcoded.

**932953.1 *Leucaniaoregona*** – This species was segregated from *Leucaniafarcta* (Grote, 1881) by Lafontaine and Schmidt (2010) and recognized as a valid species because of significant differences in the genitalia. Barcode results indicate it is closely related to *Leucaniaimperfecta* Smith, 1894, and the genitalia confirm this association, so we give it a new sequence number to reflect its proper position within *Leucania*.

**932953.2 *Leucaniachejela*** – This Caribbean and Central American species was discovered at Bahia Honda State Park in the Florida Keys by Jim Troubridge in 2013. Specimens have been dissected and barcoded.

**932955.1 *Leucaniarawlinsi*** – This species was described from Jamaica, but extends as far north as Cuba, the Bahamas, and recently was collected at the National Key Deer Refuge in the Florida Keys by Jim Troubridge. The specimen has been barcoded.

**932961.1 *Leucanialatiuscula*** – This species was described from Cuba and was collected at the National Key Deer Refuge in the Florida Keys by Jim Troubridge. A specimen has been barcoded.

**933115.1 *Rhabdorthodespattersoni*** – The genus *Rhabdorthodes* and the three constituent species are newly described in the current volume by Crabo (2018).

**933181.1 *Hypotrixlactomellis*** – This species is described in the current volume by Crabo et al. (2018).

**933207 *Hydroeciodesserrata*** – *H.ochrimacula*, **syn. rev.** does not differ from *H.serrata* in structural characters or barcodes, so we consider it to be a form of *H.serrata* and treat it as a synonym.

**933663.1 *Abagrotisbenjamini*** – This taxon was described as a “race” of *Abagrotiscrumbi*. It was raised to a valid species by Goldstein and Nelson (2017).
